# To pull or push? Performance of four passerine species in the lever-pulling task

**DOI:** 10.1007/s10071-026-02072-2

**Published:** 2026-06-02

**Authors:** Anita Szabó, Alice Exnerová

**Affiliations:** Department of Zoology, Faculty of Science, Charles University, Viničná 1594/7, Prague, 12844 Czech Republic

**Keywords:** Problem-solving, Innovative behaviour, Avian cognition, Accuracy, Persistence

## Abstract

In recent years, avian cognition has become a subject of intensive research. Most studies have, nevertheless, only focused on a few bird taxa known for their high cognitive capacities and comparative studies of other species are scarce. Testing problem-solving abilities of birds can give us an insight into how they can adapt to changing environments through innovation. Lever-pulling is a well-known paradigm to investigate problem-solving in various species, and analysing subjects’ behaviour leading to solving the task could give us important insights into the cognitive mechanisms and motor propensities potentially affecting success. Although blue tits (*Cyanistes caeruleus*) and great tits (*Parus major*) are known for successful performance during various cognitive tasks, other small passerine species inhabiting similar environments remain understudied. In contrast to great tits and blue tits, coal tits (*Periparus ater*) and nuthatches (*Sitta europaea*) are food-storing species, thus interspecific comparisons could reveal whether different foraging strategies are linked to problem-solving performance. We tested wild-caught blue and great tit adults and hand-reared juveniles of all four species in a food-motivated lever-pulling task. Results indicate species-specific differences among juveniles, with coal tits and nuthatches demonstrating greater success. Birds from both age categories performed similarly, but juveniles were faster to solve the task than adults. Success was positively influenced by individual traits such as persistence, accuracy and motivation from prior experience, but not by the initial latency to approach the apparatus. Our results reveal how both foraging strategy and individual traits could be related to innovative problem-solving in small passerines.

**Supplementary Information:**

The online version contains supplementary material available at 10.1007/s10071-026-02072-2.

## Introduction

Avian cognition has been the topic of extensive research in recent years, with an aim to uncover the mechanisms underpinning the exceptional cognitive skills of some bird species, that enable them to perform similarly to primates (e.g. Emery and Clayton 2004; Kabadayi et al. 2016; Lambert et al. 2019; Sulikowski 2021) or even outperform them in some tasks (e.g. Wright et al. 2017). Brain volume provides the structural architecture for complex computation (Pietschnig et al. 2022; Barron and Mourmourakis 2024). Specifically, larger executive regions are associated with lower metabolic costs when solving novel cognitive tasks (Jung and Haier 2007; Neubauer and Fink 2009). Several studies have linked brain size to performance in cognitive tasks (e.g. Sol et al. 2005; Deaner et al. 2007; Kabadayi et al. 2016; Audet et al. 2024). Although birds have a much smaller absolute brain size than primates, Olkowicz et al. (2016) found that the number of neurons in the forebrain of songbirds and parrots is similar to that of primates, which could explain how birds’ performance can be comparable to primates in some cognitive tasks (reviewed in Herculano-Houzel 2017). Furthermore, both absolute and relative brain size were found to positively correlate with problem-solving skills across bird species (Kabadayi et al. 2016; Audet et al. 2024).

Problem-solving ability is an important aspect of cognition and is often used as a measure for innovative ability. In problem-solving experiments, subjects usually have to interact with an apparatus and/or overcome a barrier to reach the reward, which could be either food or another resource (e.g. Keagy et al. 2009). To succeed in these tasks, animals have to innovate by either exhibiting new behaviours or using existing behaviours in a novel context (Reader and Laland 2003). In nature, problem-solving ability can allow animals to adjust to changing environments (Sol et al. 2005), for example, by finding new food sources or avoiding unfamiliar predators. Problem-solving ability can enable animals to inhabit various environments and survive even when resources are scarce, thus this ability could contribute to the fitness of the individual. For instance, Cauchard et al. (2013, 2017) found that problem solving performance of great tits (*Parus major*) is correlated with reproductive success in the wild as solver pairs fledged more young compared to non-solvers (but see Grunst et al. 2020). Furthermore, solving speed positively correlated with clutch size, hatching success and fledgling number (Cauchard et al. 2013, 2017). Cole et al. (2012) found similar results in another population of great tits using a different setup. Another study found that male satin bowerbirds (*Ptilonorhynchus violaceus*), which succeeded in a problem-solving task had higher mating success (Keagy et al. 2009).

Several studies have tested problem solving abilities of various animal taxa from leaf-cutter ants (*Atta colombica* – Dussutour et al. 2009) to birds (Lambert et al. 2019) and mammals (Benson-Amram et al. 2016). Studying problem solving ability in different species can give us an insight into cognitive evolution and species-specific differences in cognitive abilities. Although there is a continuously growing body of research on avian cognition, studies most often focus on bird taxa known for their exceptional cognitive abilities, such as corvids and parrots and other taxa are rather underrepresented in comparison (Kaplan 2015; Ten Cate and Healy 2017). Passerines are a diverse group of birds inhabiting a wide range of habitats, making them highly adaptable, which suggests an important role for innovation and problem-solving skills. Furthermore, they often live in complex social groups or forage in large flocks, giving them the opportunity to acquire novel foraging techniques by watching conspecifics or heterospecifics. For instance, milk-bottle opening in great tits and blue tits (*Cyanistes caeruleus*) began with individual innovation and subsequently spread across Great Britain as naïve individuals acquired the technique through social learning (Fisher and Hinde 1949). Comparative studies in a wider variety of bird taxa are important to better understand the drivers of cognitive evolution, and passerines represent promising candidates for problem-solving tasks.

Lever-pulling is a well-known task used for studying problem-solving abilities and several studies have tested small passerines (Morand-Ferron et al. 2011; Prasher et al. 2019; Cooke et al. 2021). In this task, subjects are presented with a transparent vertical tube containing a reward inside. A horizontal lever is supporting a small platform on which the reward is positioned, thus if the subject removes the lever, the reward drops out of the tube and becomes available. Previous studies have found that the cognitive mechanisms underlying success in this task – as well as solving similar problem-solving tasks (e.g. string-pulling) involve both operant conditioning and perceptual-motor feedback (Taylor et al. 2010; Overington et al. 2011; Cole et al. 2012). Furthermore, Cauchard et al. (2024) found that accuracy (i.e. the proportion of contacts with the part of the apparatus relevant for solving the task) had an effect on solving success as it was not only higher for solvers before initial success, but solvers also increased their accuracy over successive trials. In addition to cognitive abilities, several other factors could contribute to inter-individual variability in performance. For instance, age can be an important factor influencing success for lever-pulling, however results are contradictory. Morand-Ferron et al. (2011) found that juvenile great tits and blue tits were more likely to solve the task than adults. In contrast, Prasher et al. (2019) reported that older black-capped chickadees (*Poecile atricapillus*) outperformed younger birds. These differences could arise as younger birds tend to be more curious and less neophobic (Biondi et al. 2010), thus more willing to interact with novel objects compared to adults. However, older individuals possess more foraging experience and dexterity which could help them solve a novel problem (Thornton and Samson 2012; Griffin et al. 2014). Furthermore, personality has been suggested to influence problem-solving ability: exploratory behaviour and persistence are often found to play an important role in finding the solution to a novel problem (Overington et al. 2011; Cauchard et al. 2024). However, these factors did not predict success in lever-pulling tasks (Cole et al. 2011; Cooke et al. 2021). Although Prasher et al. (2019) found that the propensity for spatial exploration had a positive effect on the lever-pulling performance of black-capped chickadees, it was the least important predictor of success in the task. While previous research on small passerines has linked individual differences in problem-solving performance to innovative foraging ability (Cole et al. 2011), age (Morand-Ferron et al. 2011), previous experience (Cooke et al. 2021), personality (exploratory behaviour and persistence) and accuracy (Cauchard et al. 2024), studies including interspecific comparisons are essential to identify species-specific problem-solving strategies.

The first goal of this study was to find out whether species-specific strategies play a role in lever-pulling performance and to test whether food-storing and non-storing species exhibit differences in problem-solving performance outside the context of caching food. We tested four small passerine species: great tits, blue tits, coal tits (*Periparus ater*), and Eurasian nuthatches (*Sitta europaea*) in a lever-pulling task. Tits and nuthatches are small, non-migratory birds that exhibit a variety of feeding strategies and are known for their innovative behaviour (e.g. Fisher and Hinde 1949; Lefebvre 1995; Morand-Ferron et al. 2011) including occasional tool using (Morse 1968; Duyck and Duyck 1984; Shumaker et al. 2011; Rutz and Deans 2018). Furthermore, great tits and blue tits have been shown to possess various cognitive skills such as motor inhibitory-control (Urhan et al. 2023), problem-solving ability (Cole et al. 2011; Cauchard et al. 2024) and episodic-like memory (Davies et al. 2024). In contrast to great tits and blue tits, coal tits and nuthatches are food-storing species. Food-storers possess a relatively larger hippocampus compared to non-storers (Krebs et al. 1989; Sherry et al. 1989) and hippocampal size has been shown to vary across species in relation to the degree of storing (Pravosudov and Clayton 2002; Lucas et al. 2004; Gould et al. 2013). Several studies have compared food-storing and non-storing species in different cognitive tasks and found that while food-storing species perform better in tasks involving spatial memory and closely related to caching (i.e. whether caches have been emptied, what type of food they have stored and whether a potential pilferer had watched the caching event – Sherry 1984; Pravosudov 2008), non-storers could rely more on observational and social learning (i.e. watching a conspecific or a heterospecific solve a task or cache food at a specific location – Sasvári 1979; Urhan et al. 2017). In a recent review, Brodin (2025) suggested that since it is energetically costly to maintain a large brain volume, small passerines such as parids would benefit from a trade-off in cognitive abilities depending on their ecological adaptations. Therefore, food-storing parids may be specialized for spatial memory whereas non-storers would be more successful innovators and better at observational learning. However, to our knowledge there have been no studies directly comparing the performance of storers and non-storers in an innovative problem-solving task. Moreover, while great tits and blue tits have been repeatedly tested in these tasks both in the wild and under laboratory conditions (Morand-Ferron et al. 2011; Cauchard et al. 2013, 2017; Grunst et al. 2020), no such studies have been published for coal tits and Eurasian nuthatches. Interspecific comparisons in birds’ performance in the lever-pulling task could shed light on whether food-storing or non-storing species perform better in problem-solving tasks. We hypothesized that non-storing species would be more successful in solving the task owing to the possible trade-off in cognitive abilities of small parids (reviewed in Brodin 2025). However, previous studies have shown that not only cognitive abilities, but also other factors, such as the diversity of motor actions can influence success in problem-solving tasks (Griffin et al. 2014) and affect innovative foraging ability (Diquelou et al. 2016). Besides interspecific comparisons, we wanted to investigate whether age-related differences play a role in problem-solving performance by testing both wild-caught adults and hand-reared juveniles. Since younger birds tend to be more explorative and less neophobic (Biondi et al. 2010), we expected juveniles to be more successful than adults.

The second goal of this study was to disentangle the factors influencing success in the lever-pulling task. We analysed each individual’s interactions with the apparatus to understand which factors contributed to individual differences in performance in this task. We assessed time spent close to the apparatus as a measure of persistence (i.e. how long the birds spent inspecting and interacting with the apparatus), the latency to approach the apparatus for the first time as a measure of neophobia, the proportion of time spent interacting with the relevant part of the apparatus (i.e. lever) as a measure of accuracy, and whether the birds showed a preference for specific methods (i.e. push vs. pull the lever) when solving the task. Comparing solvers’ and non-solvers’ behaviour while trying to solve the task can help us understand the cognitive mechanisms underlying success in the lever-pulling task and identify other factors contributing to success. We hypothesized that solvers would spend more time close to the apparatus, they would approach a novel apparatus sooner and would be more likely to focus their attention on the relevant part of the apparatus compared to non-solvers.

The third goal of this study was to find out whether previous experience with the task had an effect on the birds’ behaviour the second time they are presented with the apparatus. We hypothesized that birds would learn from their previous experience making them more prone to focus on the relevant part of the apparatus (i.e. higher accuracy) and solve the task faster for the second time.

## Materials and methods

### Subjects and housing

We tested 49 great tits (*Parus major* − 24 adults and 25 hand-reared juveniles), 40 blue tits (*Cyanistes caeruleus* – 28 adults and 12 hand-reared juveniles), 12 hand-reared juvenile coal tits (*Periparus ater*) and 12 hand-reared juvenile nuthatches (*Sitta europaea*) during the years 2022–2023. Adults were captured using mist nets in the Botanical Garden of Charles University in Prague, Czech Republic (50.071389 N, 14.421760 E) over the year except for the breeding season (March to September). The Botanical Garden is located in the centre of the city, thus it can be characterized as an urbanised area. Upon capture, birds were sexed and aged differentiating between two age categories: yearlings and adults (Svensson 1992). The sex ratios of tested adults were: 7 male and 17 female great tits and 14 male and 14 female blue tits. After capture, birds were allowed to habituate to their home cages for 2–5 days before the beginning of the experiment. They received a diet consisting of mealworms (larvae of *Tenebrio molitor*) and commercial food mixtures: Gold Patee, Insect Patee, Uni Patee (Versele-Laga), and Expert Egg Food (Witte Molen). Adults were housed individually but in visual and acoustic contact with other birds in the aviary. Juveniles were captured in three localities in the Czech Republic: two in Prague (50.123472 N, 14.425253 E and 50.062580 N, 14.354799 E) and one in Hradec Králové (50.168989 N, 15.900398 E), all of which are large parks at the outskirts of the cities, thus while representing more natural woodland habitats, they are still located within urbanised areas. At the age of 12–15 days, birds were removed from their nest boxes and hand-reared in artificial nests on a diet of mealworms and the commercial mixture for hand-rearing birds (Handmix (Versele-Laga)) with added vitamins (Roboran). After fledging, the birds were housed in cages in groups of 2–4 individuals together with their siblings, and in visual and acoustic contact with other birds in the aviary. When the juveniles started to self-feed, they were given the same diet as adult birds. They were tested when at least 35 days old and fully independent. Thus, all juveniles spent approximately a month in the aviaries before being tested. We could not determine the sex of the juvenile birds as they were tested when they were still in their juvenile plumage, thus it was not possible to distinguish between males and females using plumage characteristics. After the experiment, all the birds were ringed and released to the locality of their origin.

### Experimental setup

Experiments were conducted either in smaller cages similar to the birds’ home cages or in larger experimental cages. Smaller cages were used for testing adult great tits and blue tits, and juvenile coal tits and nuthatches. Larger experimental cages were used for testing juvenile great tits and, originally, also juvenile blue tits. However, juvenile blue tits proved to be extremely shy and spent most of the time away from the apparatus the first time they were tested in the larger experimental cage. For this reason, they were retested in smaller cages similar to their home cages 2 months later. We found no effect of the type of cage the birds were tested in on solving success (Table S1). As none of the juvenile blue tits solved the task in the experimental cage and the number of contacts with the apparatus and the time spent close to the apparatus during the first time they were tested did not have an effect on later success (Table S2), we decided to include only the results of the juvenile blue tits from the second testing in smaller cages into the analysis.

Experimental cages were made of a wooden frame (70 × 70 × 70 cm) with wire-mesh side walls and a front wall made of one-way glass. The cages contained a wooden perch and a water bowl. Smaller cages were 50 × 40 × 50 cm with plastic side walls and a front wall with metal bars. The cages contained three wooden perches and a water bowl. All cages were illuminated by Biolux Combi 18 W (Osram) bulbs to simulate natural light conditions. Before each experiment, birds were deprived of food for 2 h to increase their foraging motivation. Water was accessible ad-libitum throughout the experiment. Trials were recorded using a video camera and recordings were later analysed for relevant behaviours using BORIS video analysis software (Friard and Gamba 2016).

### Lever-pulling task

**Fig. 1:**
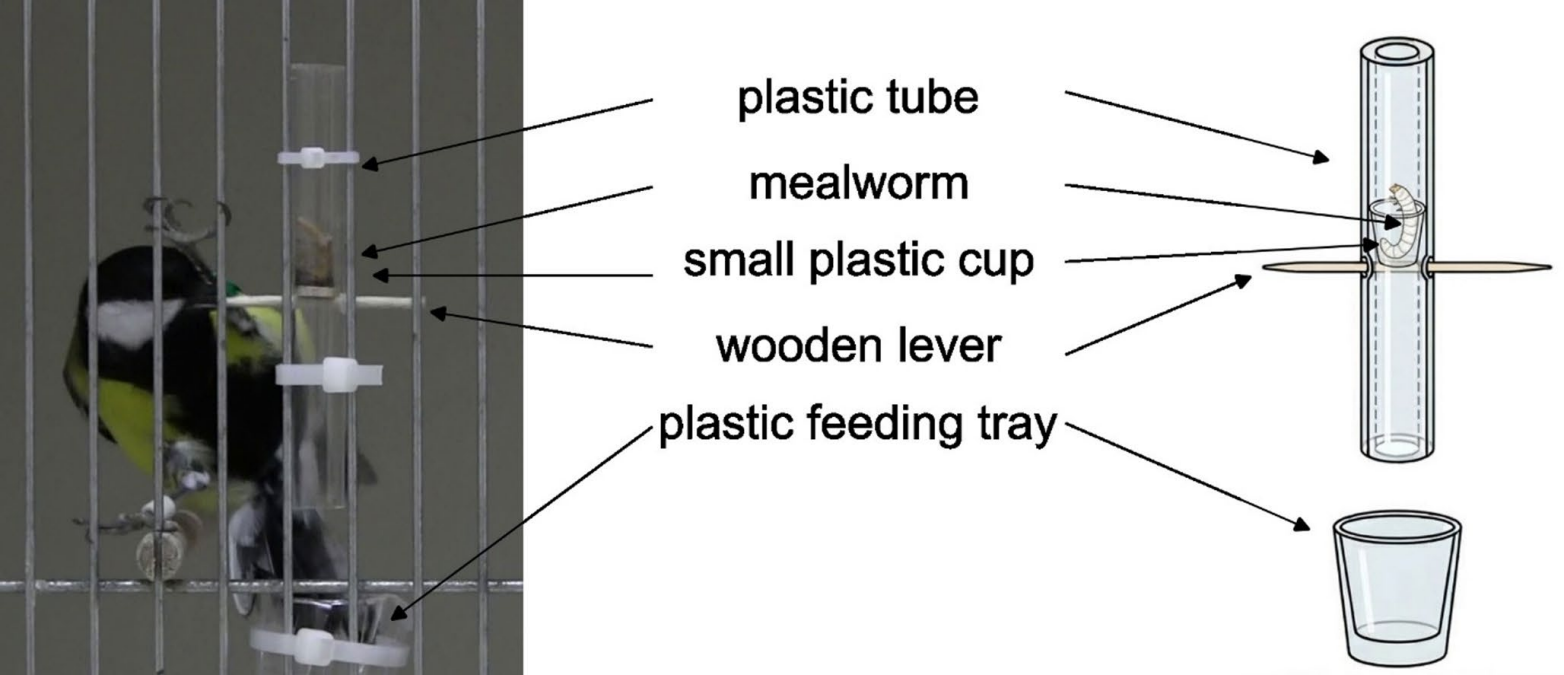
Lever-pulling apparatus in the smaller cage with a schematic drawing

The apparatus consisted of a transparent plastic tube (Plexiglas) with a diameter of 10 mm and 10 cm in length, a wooden lever (5 cm long), a circular plastic feeding tray and a small plastic cup containing one freshly moulted mealworm as a reward. The tube was attached vertically to the wire mesh/metal bars of the cage close to the wooden perch. To succeed, birds had to remove the lever, causing the plastic cup containing the reward to fall into the feeding tray (Fig. 1).

Before the experiment, birds were allowed to habituate to the experimental cage for 30–60 min, and they were offered one mealworm in the feeding tray below the apparatus to draw their attention to the apparatus. Birds received 2–4 trials. Each trial ended when a bird solved the task, or continued for a maximum of 2 h per trial. Birds that did not solve the task during the first two trials were marked as non-solvers, whereas birds that solved the task at least once during the first two trials (solvers), were allowed up to 4 attempts in total, until they reached two successful trials.

### Statistical analysis

Throughout the analysis, we split the data into two parts: great tits and blue tits dataset (both adults and juveniles to test for species and age differences) and juveniles only dataset (great tits, blue tits, coal tits, and nuthatches to test for species differences) and analysed them separately. For solvers, we only included those trials into the analysis, during which they solved the task (i.e. successful trials). Latencies were assessed only within the particular trial, not accounting for previous unsuccessful attempts. We used this approach to avoid potential differences between trials not directly related to problem-solving performance (e.g. length of inter-trial intervals, hunger level of birds, possible disturbance due to external noise). We validated this method by testing the effect of time spent close to the apparatus and the number of contacts with the apparatus in the first unsuccessful trial on later success and we found no significant effect in either of the measures (Table S1, S3). After analysing the video recordings of each trial, we categorised the birds’ behaviours and assessed the first approach latency, the proportion of time spent close to the apparatus (within body length of the bird), the mean duration of lever-pecking and tube-pecking events calculated within each trial, and whether the bird preferentially pulled the lever (1 for preference, 0 for no preference).

First, we tested the effect of species and age on solving success using a Generalized Linear Model with binomial error distribution and logit link function (GLM-b) including the species and age as predictors for the blue tits and great tits dataset and the species as predictor for the juveniles dataset. To analyse the effect of species and age on the solving latencies, we used a Cox proportional hazards model (Cox PHM) from the coxme package (Therneau 2024) with censored latencies for no events in case of non-solvers. The model included species and age as fixed effects for the blue tits and great tits dataset and the species as fixed effect for the juveniles dataset. We also used Cox PHM from the coxme package (Therneau 2024) to assess species and age differences in the latency to approach the apparatus. For the great tits and blue tits dataset, we used Wilcoxon rank-sum tests to analyse the effect of species and age separately on the proportion of time spent close to the apparatus and the mean duration of lever-pecking and tube-pecking events. For analysing species differences within the juveniles dataset in the proportion of time spent close to the apparatus and the mean duration of lever-pecking and tube-pecking events, we used Kruskal-Wallis rank sum tests with pairwise post-hoc comparisons using Wilcoxon rank sum tests. To test for species and age differences in pulling preference, we used Exact binomial tests separately for each species and age group.

Second, to assess which factors influenced success in the lever-pulling task, we used GLM-b for both datasets to test the effects of the following behaviours on solving success: the first approach latency (excluding one juvenile blue tit, which never approached the apparatus), the proportion of time spent close to the apparatus, the mean duration of lever-pecking and tube-pecking events in each trial and the preference for pulling the lever. Furthermore, we used GLM-b for both datasets to assess the effect of the type of cage the birds were tested in on solving success.

Third, to assess whether learning was involved, we used Wilcoxon signed-rank tests for both datasets to compare the birds’ behaviour between trials: the proportion of time spent close to the apparatus, the mean duration of lever-pecking and tube-pecking events in each trial and the preference for pulling the lever. Furthermore, to assess whether the above-mentioned behaviours affected success differently during the first and the second trial, we used GLM-b for each trial separately. To test for differences in pulling preference between trials, we used Exact binomial tests separately for each trial. We used Cox PHM from the coxme package (Therneau 2024) to assess the effect of trial on the latency to solve the task. The model included trial number as a fixed effect and the bird identity as a random effect.

Data analysis was performed using R version 4.4.2 (R Core Team 2020).

### Ethical note

The Ethical Committee of the Faculty of Science of Charles University provided consent to conduct the experiments. The experimental methods are included in ethical approvals issued by the Environmental Department of Municipality of Prague (MHMP-199460/2019), Ministry of Agriculture (37428/2019-MZE-18134), and Ministry of the Environment of the Czech Republic (MZP/2019/630/1082). All the birds were ringed and released at the location of capture after the experiments.

## Results

Overall, 55 birds (48.7%) succeeded in the lever-pulling task in their first trial, however since we allowed for multiple trials, 81 birds (72%) solved the task at least once and 72 birds (64%) solved it twice.

## Performance of juvenile and adult blue tits and great tits

**Fig. 2:**
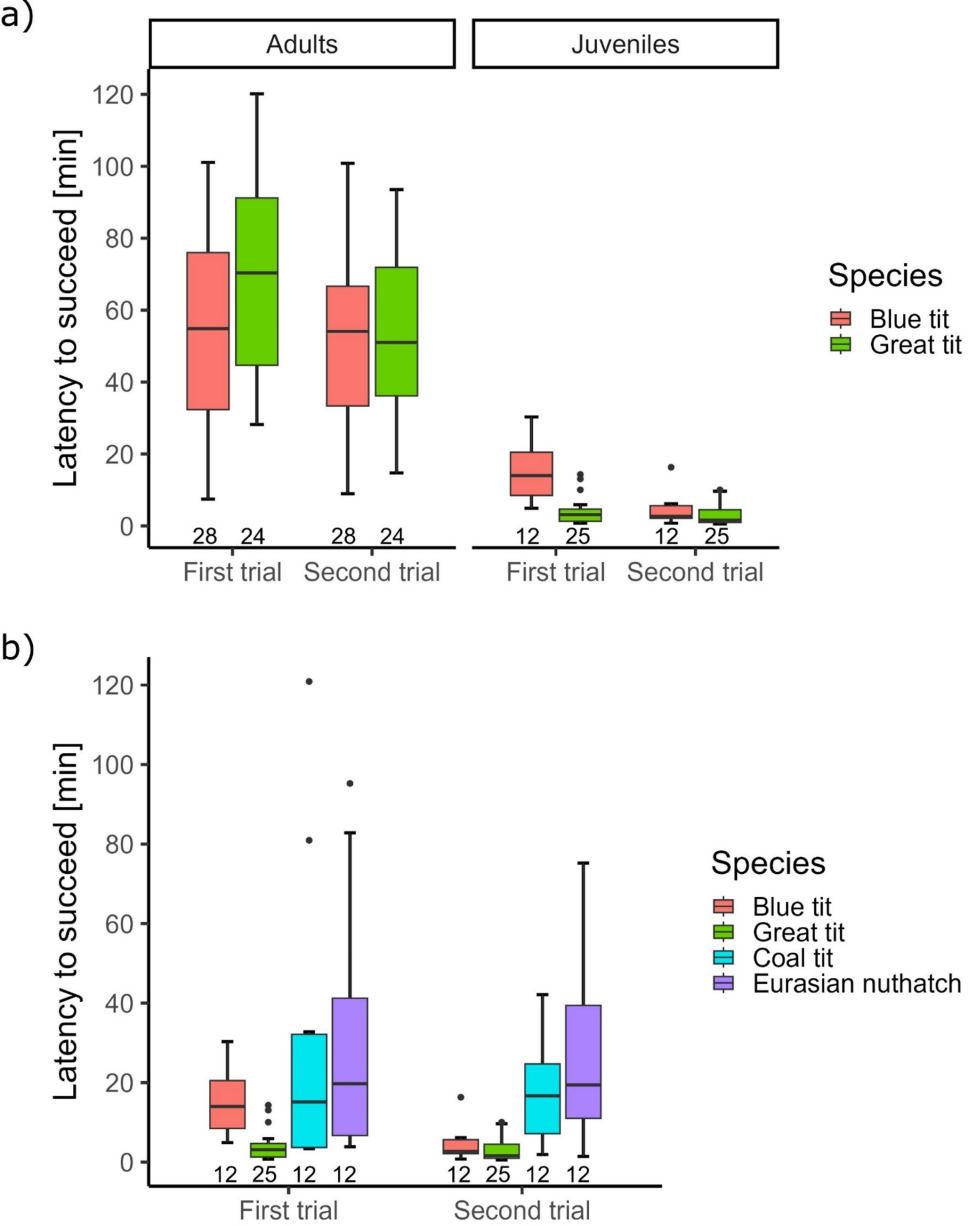
Latency to solve the task in the first and the second trial showing solvers only. (**a**) Performance of juvenile and adult blue tits and great tits. (**b**) Interspecific differences in juveniles. Horizontal lines are medians, boxes are quartiles, vertical lines show minimum and maximum values, dots represent outliers. Numbers under the boxes refer to the number of individuals tested in each category

**Fig. 3:**
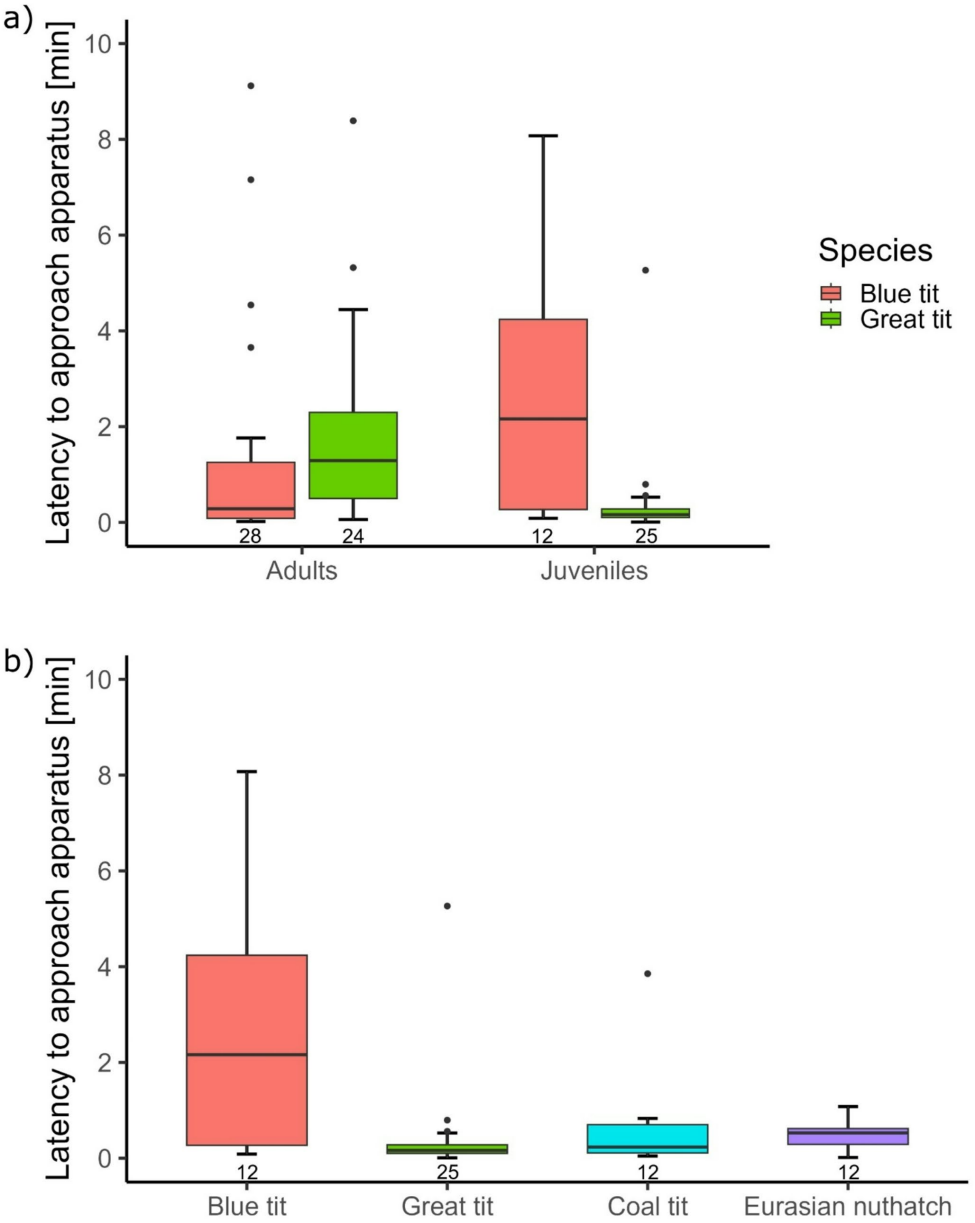
Latency to approach the apparatus for the first time showing both solvers and non-solvers. (**a**) Performance of juvenile and adult blue tits and great tits. (**b**) Species differences among juveniles. Horizontal lines are medians, boxes are quartiles, vertical lines show minimum and maximum values, dots represent outliers. Numbers under the boxes refer to the number of individuals tested in each category

Comparing the success rates between the two species and age groups, we did not find any differences (GLM-b _species_, χ^2^ = 1.24, df = 1, *p* = 0.27; GLM-b _age_, χ^2^ = 0.001, df = 1, *p* = 0.98). When comparing the latency to succeed in the task, we found an age difference with juveniles being faster than adults (Cox PHM, β = 3.07, SE = 0.79, z = 3.91, *p* < 0.001). However, there was no significant effect of species (Cox PHM, β = 0.7, SE = 0.6, z = 1.14, *p* = 0.25; Fig. 2a). Comparing the latency to approach the apparatus for the first time, we found that the interaction of species and age was significant (Cox PHM, β = 1.92, SE = 0.48, z = 4.03, *p* < 0.001). Namely, in great tits, juveniles were faster to approach the apparatus than adults (*p* < 0.001). However adult blue tits were faster than juveniles (*p* = 0.03; Fig. 3a). The latency to approach the apparatus for the first time did not have an effect on solving success (after removing one juvenile blue tit, which never approached the apparatus – GLM-b, χ^2^ = 0.76, df = 1, *p* = 0.38).

**Fig. 4:**
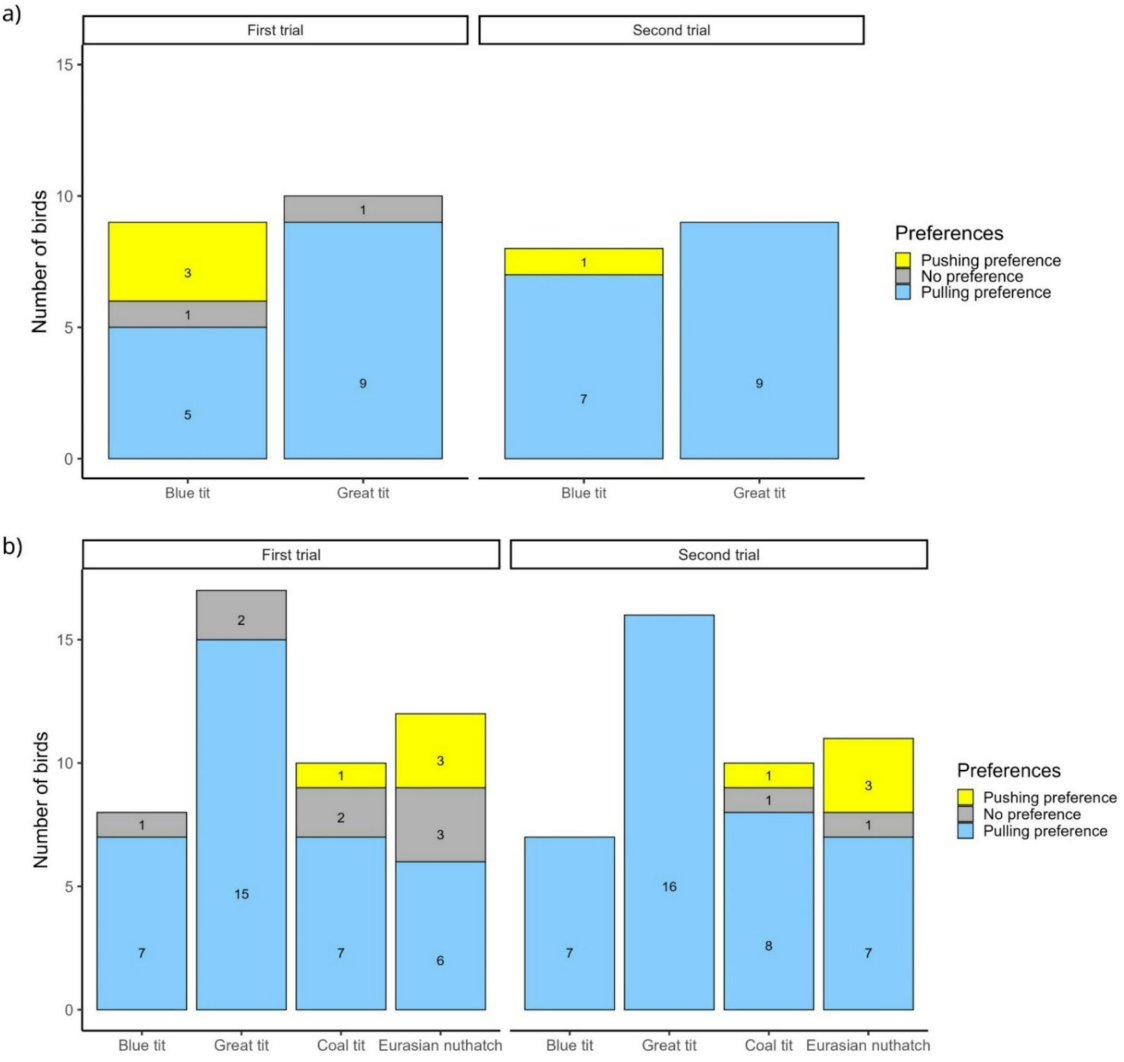
Preferences in techniques for removing the lever showing solvers only in both trials. Birds with 0-40 % preference for pulling were assigned to ‘Pushing preference’, with 40-60 % preference to ‘No preference’ and with 60-100 % preference to ‘Pulling preference’. Both graphs show the number of individuals in each category. (**a**) Species differences among adults. (**b**) Species differences among juveniles

Comparing the two different techniques for removing the lever (pulling and pushing) among solvers, we found species and age differences in the first trial. In adults, 55.5% of blue tits (binomial test, *p* = 0.5) and 90% of great tits (binomial test, *p* = 0.01) preferred pulling over pushing. This difference was not present in juveniles, as both species preferred pulling the lever (great tits: 88%, binomial test, *p* = 0.001, blue tits: 87.5%, binomial test, *p* = 0.04). During the second trial, 87.5% of adult blue tits (binomial test, *p* = 0.04), 100% of adult great tits (binomial test, *p* = 0.002) and 100% of juveniles (blue tits: binomial test, *p* = 0.008, great tits: binomial test, *p* < 0.001) preferred pulling over pushing (Fig. 4a, Table S4). The preference for pulling the lever did not have an effect on solving success during the first trial (GLM-b, χ^2^ = 0.07, df = 1, *p* = 0.8). However, during the second trial, we found a significant effect of pulling preference on solving success (GLM-b, χ^2^ = 4.09, df = 1, *p* = 0.04; Table S5).

The proportion of time spent close to the apparatus differed between species (Wilcoxon _first trial_, W = 680, *p* = 0.01; Wilcoxon _second trial_, W = 615, *p* = 0.01) and age categories (Wilcoxon _first trial_, W = 484, *p* < 0.001; Wilcoxon _second trial_, W = 631, *p* = 0.02), with great tits spending a larger proportion of time close to the apparatus (0.4 ± 0.32; 0.39 ± 0.3) than blue tits (0.2 ± 0.15; 0.23 ± 0.24) and juveniles (0.49 ± 0.34; 0.45 ± 0.34) spending longer time than adults (0.19 ± 0.12; 0.22 ± 0.19; Table S6). Moreover, the time spent close to the apparatus significantly affected success in both trials (0.32 ± 0.28; GLM-b _first trial_, χ^2^ = 14.57, df = 1, *p* < 0.001; GLM-b _second trial_, χ^2^ = 30.95, df = 1, *p* < 0.001; Table S5).

Comparing the mean duration of individual lever-pecking and tube-pecking events, we found that in the first trial, success did not depend on the mean pecking duration (lever: 1.32 ± 1.52 s; GLM-b, χ^2^ = 0.009, df = 1, *p* = 0.92, tube: 2.1 ± 1.9 s; GLM-b, χ^2^ = 3.66, df = 1, *p* = 0.06). In the second trial, the mean duration of lever-pecking (1.09 ± 1 s; GLM-b, χ^2^ = 7.28, df = 1, *p* = 0.007) and tube-pecking events (1.72 ± 1.31 s; GLM-b, χ^2^ = 6.7, df = 1, *p* = 0.01) explained solving success (Table S5) as non-solvers showed a slight decrease in the mean pecking duration of the lever (1.36 ± 1.54 s vs. 1.1 ± 1 s; Wilcoxon, V = 264, *p* = 0.07), whereas both solvers and non-solvers had shorter pecking duration of the tube compared to the first trial (2.1 ± 1.9 s vs. 1.72 ± 1.31 s; Wilcoxon, V = 2233, *p* = 0.05).

The latency to succeed was not different between the two trials (Cox PHM, β = 0.4, SE = 0.45, z = 0.79, *p* = 0.43; Fig. 2a) and solvers did not significantly increase the proportion of time spent close to the apparatus during the second trial (0.46 ± 0.29) compared to the first one (0.39 ± 0.3; Wilcoxon, V = 535, *p* = 0.6).

Analysing the effect of sex, we only found a difference in the first approach latency between males and females (Cox PHM _sex_: β = 0.86, z = 2.12, *p* = 0.03; Cox PHM _sex*species_: β = -1.21, z = -1.99, *p* = 0.05) as blue tit males approached the apparatus faster than females (*p* = 0.03; Table S7).

## Interspecific differences in juveniles

When comparing the success rates in juveniles, we found differences among species (GLM-b, χ^2^ = 9.69, df = 3, *p* = 0.02). Namely, all nuthatches and all but one of the coal tits solved the task, whereas great tits and blue tits were less successful. The latency to solve the task was also different among the four species: great tits were faster compared to blue tits (Cox PHM, β = -1.61, SE = 0.8, z = -2.01, *p* = 0.04) and nuthatches (Cox PHM, β = -1.44, SE = 0.73, z = -1.98, *p* = 0.05; Fig. 2b). Comparing the latency to approach the apparatus for the first time, we found that blue tits took longer to approach than other species (Cox PHM, blue tits-great tits: β = 1.64, SE = 0.43, z = 3.85, *p* < 0.001; blue tits-coal tits: β = 1.2, SE = 0.5, z = 2.56, *p* = 0.01; blue tits-nuthatches: β = 1, SE = 0.48, z = 2.1, *p* = 0.04; Fig. 3b) and the first approach latency did not have an effect on solving success (after removing one juvenile blue tit, which never approached the apparatus – GLM-b, χ^2^ = 1.34, df = 1, *p* = 0.25). Comparing the two different techniques for removing the lever (pulling and pushing) among solvers in the first trial, we found species differences, namely, 87.5% of blue tits (binomial test, *p* = 0.04) and 88% of great tits (binomial test, *p* = 0.001) preferred pulling, whereas only 70% of coal tits (binomial test, *p* = 0.17) and 50% of nuthatches (binomial test, *p* = 0.61) preferentially pulled the lever. During the second trial, all three tit species preferentially pulled the lever (blue tits: 100%, binomial test, *p* = 0.008, great tits: 100%, binomial test, *p* < 0.001, coal tits: 80%, binomial test, *p* = 0.05). However, nuthatches did not show a significant preference (64%, binomial test, *p* = 0.27) for this technique (Fig. 4b, Table S8).

The preference for pulling the lever did not have an effect on solving success neither during the first (GLM-b, χ^2^ = 1.15, df = 1, *p* = 0.28) nor during the second trial (GLM-b, χ^2^ = 2.66, df = 1, *p* = 0.1, Table S9).

The proportion of time spent close to the apparatus differed between species (Kruskal-Wallis, χ^2^ = 8.87, df = 3, *p* = 0.03) with great tits tending to spend a larger proportion of time close to the apparatus (0.58 ± 0.33) than blue tits (0.28 ± 0.22; *p* = 0.08) during the first trial (Table S10). Furthermore, the time spent close to the apparatus had a significant effect on solving success in both trials (0.43 ± 0.29; GLM-b _first trial_, χ^2^ = 13.8, df = 1, *p* < 0.001; GLM-b _second trial_, χ^2^ = 30.33, df = 1, *p* < 0.001, Table S9).

Comparing the mean duration of lever-pecking and tube-pecking events, we found similar trends for both, namely that for the first trial, there was no effect of the mean duration on solving success (lever: 1.78 ± 1.24 s; GLM-b, χ^2^ = 1.69, df = 1, *p* = 0.19, tube: 2.55 ± 2.01 s; GLM-b, χ^2^ = 1.89, df = 1, *p* = 0.17). However, for the second trial, the mean duration of pecking events had a significant effect on solving success (lever: 1.67 ± 1.15 s; GLM-b, χ^2^ = 12.14, df = 1, *p* < 0.001, tube: 1.82 ± 1.14 s; GLM-b, χ^2^ = 13.28, df = 1, *p* < 0.001, Table S9). The two trials differed because non-solvers spent less time pecking the lever in the second trial (1.69 ± 1.39 s vs. 1.45 ± 1.08 s), although the difference was not significant (Wilcoxon, V = 56, *p* = 0.2), while both solvers and non-solvers decreased their time spent tube pecking in the second trial (1.82 ± 1.14 s) compared to the first one (2.55 ± 2.01 s; Wilcoxon, V = 1213, *p* = 0.01).

The latency to succeed did not differ between the two trials (Cox PHM, β = 0.22, SE = 0.4, z = 0.56, *p* = 0.58; Fig. 2b). We did not find a difference in the time spent by solvers close to the apparatus between the two trials (0.45 ± 0.28 vs. 0.47 ± 0.28; Wilcoxon, V = 433, *p* = 0.24).

## Discussion

The overall success rate of birds in our experiment (48.7%) was similar to that of other studies of great tits and black-capped chickadees (44% in Cole et al. 2011; 42% in Quinn et al. 2016; 54% in Prasher et al. 2019) and we found no difference in solving success between adults and juveniles, which is also in accordance with previous research (Cole et al. 2011; Griffin et al. 2014; but see Biondi et al. 2010; Morand-Ferron et al. 2011). However, our results show that juveniles were faster to succeed than adults, which is in line with findings by Griffin et al. (2014). One explanation for this could be that although juveniles are usually more curious and less neophobic (Biondi et al. 2010), thus more prone to interact with novel objects, adults might possess more dexterity due to their foraging experience, which could aid them in solving a novel problem (Thornton and Samson 2012; Griffin et al. 2014). Although our juveniles were hand-reared, thus they were more familiar with cages than the wild-caught adults, the lever-pulling apparatus was a novel device for both age groups, and we did not find any differences in the latency to approach the apparatus between adults and juveniles. However, we found that juveniles spent a larger proportion of time close to the apparatus compared to adults, which had an effect on successfully solving the task. Therefore, we believe, that the differences in the latency to succeed between the hand-reared juveniles and wild-caught adults could be attributed mainly to curiosity and increased interest of the juveniles in a novel apparatus and less so to the differences in rearing conditions. Overall, our birds did not solve the task faster in the second successful trial when they were already experienced, which is similar to what Cook et al. (2017) found in juvenile house finches (*Haemorhous mexicanus*).

We found species-specific differences in success rate among our hand-reared juveniles (blue tits: 67%, great tits: 68%, coal tits: 92%, nuthatches: 100%): coal tits and nuthatches were more successful and they did not show a significant preference for pulling the lever in the first successful trial but tried both pulling and pushing to succeed. Previous studies have found that motor diversity influences the propensity to succeed in a problem-solving task (Griffin et al. 2014) and to forage innovatively (Diquelou et al. 2016). Therefore, it is possible, that as both coal tits and nuthatches are food-storing species, they might need to exhibit various techniques to hide and retrieve food items (e.g. wedging the piece of food into crevices in the bark of a tree), which could make them more prone to find solutions to novel problems that are related to food-storing behaviour. However, so far there is no evidence for food-storing species to exhibit increased motor diversity compared to other species.

Interestingly, in the first trial, the mean duration of pecks on the tube and on the lever were not different between solvers and non-solvers, however in the second trial, non-solvers decreased the mean pecking time on the whole apparatus (i.e. both the lever and the tube) whereas solvers only decreased pecking time on the irrelevant part of the apparatus (i.e. the tube). Thus, it seems that solvers learned which part of the apparatus is relevant for receiving the reward, whereas non-solvers lost interest in the whole apparatus after the unsuccessful first trial. This finding is in line with other studies showing that accuracy (i.e. interacting with the relevant part of the problem-solving apparatus) has an effect on solving success (Cauchard et al. 2024) and positive feedback through previous experience can also influence problem-solving performance (Cooke et al. 2021). We also found that the proportion of time spent close to the apparatus predicted solving success independent of species or age. These findings indicate that the birds might have succeeded by persistently interacting with the apparatus until they solved the task (i.e. trial-and-error learning) and not by focusing on a particular part of the apparatus at first or by using a certain technique. This could provide us with another possible explanation as to why our hand-reared coal tits and nuthatches performed better than great tits and blue tits in this task. It is possible that while blue tits and great tits are better at observational learning, they might require longer time to innovate and find the solution to a novel problem, whereas coal tits and nuthatches could be faster at solving the task by trial-and-error learning owing to their food-storing behaviour and the diversity of motor actions linked to this foraging strategy. Thus, while our results are contrary to our expectations based on a possible trade-off in cognitive abilities of food-storing and non-storing species (reviewed in Brodin 2025), our findings indicate that there might be other factors – the diversity of motor actions (i.e. pulling and pushing the lever) and the possibility for trial-and-error learning – influencing success in a problem-solving task, and differences in cognitive abilities might not account entirely for interspecific differences in performance. However, since we only tested hand-reared juveniles, it remains to be investigated whether adult coal tits and nuthatches are more successful in problem-solving tasks than great tits and blue tits.

Moreover, we found interspecific differences in the latency to first approach the apparatus, with juvenile blue tits taking longer to approach compared to the other three species. Additionally, we found opposite trends in how latencies to approach the apparatus differed for juvenile versus adult great tits and blue tits, as great tits showed a tendency to be more cautious as adults whereas blue tits were more cautious as juveniles. Blue tits have been shown to exhibit innate neophobia towards novel prey (Exnerová et al. 2007) and it has been found that, unlike great tits and coal tits, juvenile blue tits remain cautious with novel prey regardless of their previous experience (Adamová-Ježová et al. 2016). Although our experiment did not involve novel food, it is possible that blue tits are more cautious and neophobic than similarly sized tit species in other contexts as well, however this remains to be investigated. The latency to approach the apparatus did not influence solving success similarly to previous studies (Cole et al. 2011; Audet et al. 2024; Cauchard et al. 2024).

Although our results are in line with those found in previous studies, we have to interpret our findings with caution. First of all, we found individual differences with several outliers in most of the investigated measures and we did not have the possibility to test the same number of individuals for each species and age group. Since in two of the species studied, we only tested hand-reared juveniles, further studies are needed to verify whether the interspecific differences in solving the lever-pulling task are also present in adults. Since we tested both wild-caught adults and hand-reared juveniles, we could not standardise the time of the year for the experiment as adults were only available outside of the breeding season and juveniles could be tested only during the summer months. We tried to reduce the differences in body condition due to environmental factors by keeping each bird in the aviary several days before the start of the experiments with highly nutritional food available at all times. Furthermore, a study by Morand-Ferron et al. (2011) confirmed that success in a problem-solving task in captivity did not predict success in the wild, thus they concluded that the propensity to innovate not only depends on individual differences but also on the context. Thus, follow-up studies including field experiments are necessary to fully assess problem-solving abilities of birds in the wild. Besides the context, there are other factors which could influence the results. For instance, the nature of the task itself can lead to different outcomes as Preiszner et al. (2017) found that individual performance did not correlate between an obstacle-removal and a food-acquisition task and the authors argued that the difference in performance between the two tasks could be attributed to differences in motivation to solve the task. Similarly, Prasher et al. (2019) found that the characteristics of the task, food deprivation and the visibility of the food reward can have an effect on solving performance. Furthermore, a study by Vincze et al. (2024) pointed out that differences in the experimental setup, such as the size and material of the apparatus can influence problem-solving performance even when subjects are presented with otherwise identical tasks. Therefore, repeating this study using different tasks could give us important insights into interspecific differences in problem-solving abilities. In conclusion, our study brings novel insights into the behaviours influencing success in a lever-pulling task while comparing the performance of adults and juveniles and four species with different ecological traits. Although several studies have tested problem-solving performance in birds, comparative studies with unified methodologies and more field experiments are needed to truly understand the cognitive abilities of different species.

## Conclusions

The two food-storing species (coal tits and nuthatches) were highly successful in the lever-pulling task and juvenile blue tits and great tits were faster to solve the task than adults of the same species. The first approach latency did not have an effect on solving success, whereas solvers spent more time close to the apparatus than non-solvers. The time spent pecking the lever and the tube did not influence success in the first trial, however during the second trial, solvers selectively decreased pecking the tube indicating that they learned which part of the apparatus is relevant for successfully solving the task. Comparison of the two techniques for removing the lever revealed that coal tits and nuthatches did not show a clear preference for any one technique at first. However, coal tits preferentially pulled the lever once they were experienced with solving the task.

## Supplementary Information

Below is the link to the electronic supplementary material.

Supplementary Material 1

## Data Availability

Data is available on Figshare under the following identifier: 10.6084/m9.figshare.32524761.
