# Supplementary material for: To pull or push? Performance of four passerine species in the lever-pulling task
Source: Anim Cogn. 2026 Jun 2;29(1):53. doi: 10.1007/s10071-026-02072-2 (PMC13454000; doi:10.1007/s10071-026-02072-2)
Supplement: Supplementary file 1 [file 10071_2026_2072_MOESM1_ESM.pdf]

To pull or push? Performance of four passerine species in the lever-pulling task

Animal Cognition

Anita Szabó[1]*, Alice Exnerová[1]

[1]Department of Zoology, Faculty of Science, Charles University, Prague, Czech Republic

*Corresponding author: Department of Zoology, Faculty of Science, Charles University, Viničná 1594/7, Prague 2, 12844, Czech Republic.

Email: szaboan@natur.cuni.cz

Table S1 Effect of six variables measured during the first unsuccessful trial on later success from (a) the great tits and blue tits dataset and (b) the juveniles dataset. The tables show results of GLM-b for each variable.

(a)

**Variable**	**χ^2**	**df**	**p**
Type of cage	0.9	1	0.34
Number of contacts with apparatus	0.003	1	0.96
Proportion of time spent close	0.002	1	0.96
First approach latency	0.0001	1	0.99
Mean duration of lever-pecking	0.03	1	0.87
Mean duration of tube-pecking	0.25	1	0.62

(b)

Variable	χ^2	df	p
Type of cage	0.29	1	0.59
Number of contacts with apparatus	0.96	1	0.33
Proportion of time spent close	0.0006	1	0.98
First approach latency	0.48	1	0.49
Mean duration of lever-pecking	0.07	1	0.8
Mean duration of tube-pecking	0.88	1	0.35

Table S2 Effect of two measured variables in the first unsuccessful trial (i.e. in the experimental cage) by juvenile blue tits on later success in the lever-pulling task. The table shows results of GLM-b for each variable.

Variable	χ^2	df	p
Number of contacts with apparatus	0.59	1	0.44
Proportion of time spent close	2.58	1	0.11

Table S3 Difference in four measured variables between the first unsuccessful and the first successful trial from (a) the great tits and blue tits dataset and (b) the juveniles dataset. The tables show results of Wilcoxon signed rank tests for each variable.

(a)

Variable	V	p
First approach latency	111	0.11
Proportion of time spent close	37	0.06
Mean duration of lever-pecking	60	1
Mean duration of tube-pecking	83	0.78

(b)

Variable	V	p
First approach latency	42	0.16
Proportion of time spent close	4.5	0.02
Mean duration of lever-pecking	16	0.5
Mean duration of tube-pecking	17	0.32

Table S4 Species and age differences in the preference for pulling the lever from the great tits and blue tits dataset during the (a) first and the (b) second trial. Both tables show the percentage of individuals with a preference for pulling and the significance values based on Binomial tests.

a)

Age/ Species	Blue tit	Binomial test	Great tit	Binomial test
Adults	55.50%	$p = 0.5$	90%	$p = 0.01$
Juveniles	87.50%	$p = 0.04$	88%	$p = 0.001$

b)

Age/ Species	Blue tit	Binomial test	Great tit	Binomial test
Adults	87.50%	$p = 0.04$	100%	$p = 0.002$
Juveniles	100%	$p = 0.008$	100%	$p < 0.001$

Table S5 Effect of four measured variables on success from the great tits and blue tits dataset (both adults and juveniles) during the (a) first and the (b) second trial. Both tables show means, standard deviations, and results of GLM-b for each variable.

a)

Variable	Mean	SD	χ^2	df	p
Proportion of time spent close	0.32	± 0.27	14.57	1	< 0.001
Mean duration of lever-pecking	1.32 s	± 1.52 s	0.009	1	0.92
Mean duration of tube-pecking	2.1 s	± 1.9 s	3.66	1	0.06
Preference for pulling	-	-	0.07	1	0.8

b)

Variable	Mean	SD	χ^2	df	p
Proportion of time spent close	0.32	0.28	30.95	1	< 0.001
Mean duration of lever-pecking	1.09 s	± 1 s	7.28	1	0.007
Mean duration of tube-pecking	1.72 s	± 1.31 s	6.7	1	0.01
Preference for pulling	-	-	4.09	1	0.04

Table S6 Effect of species and age on three measured variables from the great tits (GT) and blue tits (BT) dataset (both ad: adults and juv: juveniles) during the (a) first and the (b) second trial. Both tables show means, standard deviations, and results of Wilcoxon rank-sum tests for each variable.

a)

	Effect of species				Effect of age			
**Variable**	**Mean**	**SD**	**W**	**p**	**Mean**	**SD**	**W**	**p**
Proportion of time spent close	GT: 0.4<br>BT: 0.2	GT: ± 0.32<br>BT: ± 0.15	680	0.01	juv: 0.49<br>ad: 0.19	juv: ± 0.34<br>ad: ± 0.12	484	< 0.001
Mean duration of lever-pecking	GT: 1.12 s<br>BT: 1.57 s	GT: ± 0.93 s<br>BT: ± 1.99 s	1005	0.84	juv: 1.48 s<br>ad: 1.21 s	juv: ± 1.27 s<br>ad: ± 1.66 s	706	0.03
Mean duration of tube-pecking	GT: 2.61 s<br>BT: 1.47 s	GT: ± 2.25 s<br>BT: ± 1.02 s	569	< 0.001	juv: 2.85 s<br>ad: 1.56 s	juv: ± 2.41 s<br>ad: ± 1.14 s	565	< 0.001

b)

	Effect of species				Effect of age			
**Variable**	**Mean**	**SD**	**W**	**p**	**Mean**	**SD**	**W**	**p**
Proportion of time spent close	GT: 0.39<br>BT: 0.23	GT: ± 0.3<br>BT: ± 0.24	615	0.01	juv: 0.45<br>ad: 0.22	juv: ± 0.34<br>ad: ± 0.19	631	0.02
Mean duration of lever-pecking	GT: 0.96 s<br>BT: 1.27 s	GT: ± 0.8 s<br>BT: ± 1.2 s	994	0.45	juv: 1.19 s<br>ad: 1.02 s	juv: ± 0.86 s<br>ad: ± 1.09 s	738	0.14
Mean duration of tube-pecking	GT: 1.74 s<br>BT: 1.7 s	GT: ± 1.22 s<br>BT: ± 1.43 s	800	0.35	juv: 1.52 s<br>ad: 1.87 s	juv: ± 1.25 s<br>ad: ± 1.34 s	1030	0.28

Table S7 Species and sex differences in the first approach latency from the great tits and blue tits dataset. The table shows means, standard deviations and the results of Cox proportional hazards model (Cox PHM).

**Sex**	**Mean**	**SD**	**COX PHM**
Blue tit	males: 36.65 s<br>females: 117.45 s	males: ± 55.53 s<br>females: ± 167.69 s	Sex: β = 0.86, z = 2.12, p = 0.03<br>Sex*species: β = -1.21, z = -1.99, p = 0.05
Great tit	males: 149.32 s<br>females: 98.1 s	males: ± 98.63 s<br>females: ± 120.87 s	

Table S8 Species differences in the preference for pulling the lever from the juveniles dataset during the (a) first and the (b) second trial. Both tables show the percentage of individuals with a preference for pulling and the significance values based on Binomial tests.

a)

**Species**	**Pulling preference**	**Binomial test**
Blue tit	87.50%	p = 0.04
Great tit	88.00%	p = 0.001
Coal tit	70%	p = 0.17
Nuthatch	50%	p = 0.61

b)

Species	Pulling preference	Binomial test
Blue tit	100.00%	$p = 0.008$
Great tit	100.00%	$p < 0.001$
Coal tit	80%	$p = 0.05$
Nuthatch	64%	$p = 0.27$

Table S9 Effect of four measured variables on success from the juveniles dataset (great tits, blue tits, coal tits and nuthatches) during the (a) first and the (b) second trial. Both tables show means, standard deviations, and results of GLM-b for each variable.

a)

Variable	Mean	SD	χ^2	df	p
Proportion of time spent close	0.43	± 0.29	13.8	1	< 0.001
Mean duration of lever-pecking	1.78 s	± 1.24 s	1.69	1	0.19
Mean duration of tube-pecking	2.55 s	± 2.01 s	1.89	1	0.17
Preference for pulling	-	-	1.15	1	0.28

b)

Variable	Mean	SD	χ^2	df	p
Proportion of time spent close	0.45	0.29	30.33	1	< 0.001
Mean duration of lever-pecking	1.67 s	± 1.15 s	12.14	1	< 0.001
Mean duration of tube-pecking	1.82 s	± 1.14 s	13.28	1	< 0.001
Preference for pulling	-	-	2.66	1	0.1

Table S10 Effect of species on three measured variables from the juveniles dataset (GT: great tits, BT: blue tits, CT: coal tits and NH: nuthatches) during the (a) first and the (b) second trial. Both tables show means, standard deviations, and results of Kruskal-Wallis rank sum tests for each variable.

a)

Variable	Mean	SD	χ^2	df	p
Proportion of time spent close	GT: 0.58<br>BT: 0.28<br>CT: 0.35<br>NH: 0.32	GT: ± 0.33<br>BT: ± 0.22<br>CT: ± 0.22<br>NH: ± 0.15	8.87	3	0.03
Mean duration of lever-pecking	GT: 1.43 s<br>BT: 1.6 s<br>CT: 2.06 s<br>NH: 2.4 s	GT: ± 1.04 s<br>BT: ± 1.64 s<br>CT: ± 1.3 s<br>NH: ± 0.66 s	12	3	0.007
Mean duration of tube-pecking	GT: 3.31 s<br>BT: 1.89 s<br>CT: 2.21 s<br>NH: 1.98 s	GT: ± 2.65 s<br>BT: ± 1.4 s<br>CT: ± 1.12 s<br>NH: ± 0.79 s	3.01	3	0.39

b)

Variable	Mean	SD	χ^2	df	p
Proportion of time spent close	GT: 0.51<br>BT: 0.32<br>CT: 0.55<br>NH: 0.35	GT: ± 0.33<br>BT: ± 0.3<br>CT: ± 0.16<br>NH: ± 0.16	6.62	3	0.09
Mean duration of lever-pecking	GT: 1.18 s<br>BT: 1.22 s<br>CT: 2.19 s<br>NH: 2.6 s	GT: ± 0.87 s<br>BT: ± 0.83 s<br>CT: ± 1.14 s<br>NH: ± 1.12 s	14.79	3	0.002
Mean duration of tube-pecking	GT: 1.58 s<br>BT: 1.4 s<br>CT: 2.5 s<br>NH: 2.1 s	GT: ± 1.15 s<br>BT: ± 1.42 s<br>CT: ± 0.55 s<br>NH: ± 0.82 s	12.52	3	0.006
